# A practical perspective for chromatic orthogonality for implementing in photolithography

**DOI:** 10.1038/s41598-023-27869-w

**Published:** 2023-01-13

**Authors:** Godlaveeti Sreenivasa Kumar, Mizaj Shabil Sha, Swathi Yempally, John-John Cabibihan, Kishor Kumar Sadasivuni

**Affiliations:** 1 Center for Advanced Materials, Qatar University, PO Box 2713, Doha, Qatar; 2 Department of Mechanical and Industrial Engineering, Qatar University, PO Box 2713, Doha, Qatar

**Keywords:** Chemistry, Materials science, Physics

## Abstract

Theoretically, it is more challenging to anticipate the conversion and selectivity of a photochemical experiment compared to thermally generated reactivity. This is due to the interaction of light with a photoreactive substrate. Photochemical reactions do not yet receive the same level of broad analytical study. Here, we close this research gap by presenting a methodology for statistically forecasting the time-dependent progression of photoreactions using widely available LEDs. This study uses NiS/ZnO in perovskite (MAPbI_3_) solar cells as an additive (5 volume %). The effect of monolithic perovskite solar cells (mPSCs) on forecasting the wavelength of LEDs has been carefully investigated using various characterization methods, including X-ray diffraction (XRD) and Transmission electron microscopy (TEM). The photocatalytic activity was analyzed by measuring the voltage produced. Various factors like selectivity, stability and sensitivity were also examined. This work provides a new perspective to validate NiS/ZnO photocatalysts for predicting the wavelength of different light sources and to apply in photolithography.

## Introduction

Since photosynthesis, supported by energy from the sun, sustains life on Earth. Photochemistry is possibly the most extensive, significant, and enabling of all chemical processes^1^. The body of knowledge on photochemical processes is extensive and varied, and it has grown significantly since the invention of laser technology. It is now possible to precisely alter the wavelength of light in addition to its intensity^2,3^.

Photo chemists have tried to figure out which light color will provide the most efficient reaction kinetics. A molecule's absorption spectrum directs the most efficient electronic transitions on the fundamental level, frequently shown in a Jablonski diagram^4,5^. Before beginning any synthetic endeavor, the absorption spectrum can be computationally determined using quantum chemistry techniques like density functional theory (DFT). Information from the absorption spectrum alone cannot forecast the wavelength at which photochemical reaction pathways can be most successfully activated^6–8^.

Current soft-matter materials research and polymer chemistry may chemically support numerous photoactive materials. Photoresists, medicinal supplies, and surface coatings are some materials (reaction mixtures that solidify under the influence of light)^9^. In the past, these photoresists were usually cured with a specific hue of light (or a limited spectrum of colors given by an LED or, in certain situations, with extremely broad emitting light sources), producing a particular material feature^10^. Photoresist technology has recently introduced multi-component mixes with special reactive chromophores that respond to a particular light hue^11^. Nearly complete wavelength orthogonality (also known as chromatic orthogonality or chromatic selectivity) between the photoreactive groups is necessary to produce such a selective photoresist^12^. No matter the sequence of the various light hues, it isn't easy to activate the chromophores^13,14^. To achieve such a high degree of orthogonality, it is essential to understand the wavelength-resolved reactivity for each photoactive component in the solution concerning their absorption spectra in the same solvent. Numerous applications include functionalized surfaces, hydrogels and lithography, control of nucleic acids, gene expression, regulation of enzyme activity and interfering with neuronal processes^15,16^.

In the past few decades, Numerous photocatalysts have been investigated and studied. Because of its non-toxicity, high durability, high stability, and low cost, ZnO is regarded as one of the best semiconductor materials utilized in photocatalysis for hydrogen evolution, carbon dioxide utilization, and mineralization of organic pollutants^17,18^.

According to recent studies, the value of individual semiconductors is limited, and their ineffectiveness at charge separation and greater bandgaps prevent them from effectively removing contaminants^19,20^. Some dopants or metal oxides change semiconductors to boost photocatalytic activity. ZnO and NiS semiconductors have undergone various modifications to enable photocatalysis with a higher quantum efficiency of the photocatalyst. Water splitting, CO_2_ utilization, electrocatalysis, and water filtration have been the main applications for NiS-based heterostructures. NiS nanoparticles are employed as a cocatalyst to boost photocatalytic activity by creating an interfacial electric field of nanocomposites. NiS nanoparticles are chosen because they facilitate the efficient charge separation of photogenerated excitons (e−/h+) and produce many reaction sites for photocatalysis^21–23^. As a result, we anticipate that the nanocomposite NiS/ZnO will be an outstanding photocatalyst with a restricted charge recombination rate and robust redox capacity for effectively sensing various wavelengths.

We present a method to evaluate this photocatalyst's possible wavelength-dependent selectivity and direct the corresponding experiment's design. NiS/ZnO heterostructure photocatalysts were designed to achieve wavelength prediction at the expense of UV–visible light irradiation. To our knowledge, we first disclose its photocatalytic property, mostly used for chromatic orthogonality and energy harvesting.

## Results and discussion

### Morphological and structural analysis

**Figure 1:**
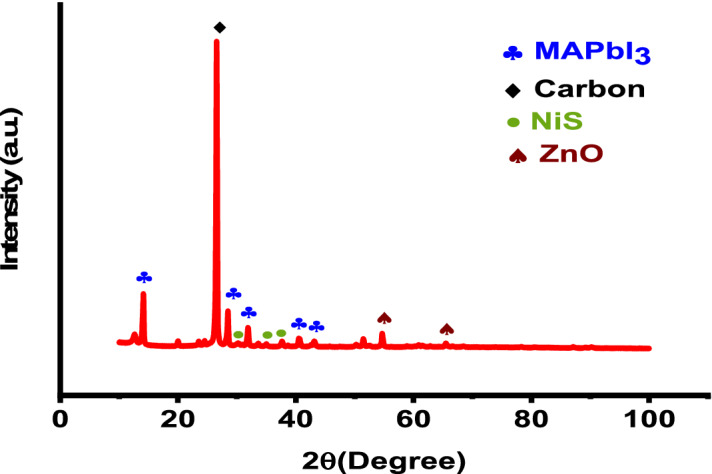
XRD of NiS/ZnO with perovskite.

XRD (Model No. EMPYREAN) plots for pure perovskite and films with 5% NiS/ZnO addition were obtained to assess the crystallinity of materials. The XRD is shown in Fig. 1. The samples detect a strong peak reflecting the (1 1 0) crystal plane of the MAPbI_3_ phase at an angle of 14.2, confirming the cubic phase of the perovskite. The (0 0 2) plane of carbon is represented by a sharp diffraction peak at 2θ = 26.2°. The distinctive diffraction peaks at angles (2θ) of 28.3, 31.6, 40.5, and 43.1 are indexed to the MAPbI_3_ reflections at (0 0 4), (2 2 0), (3 1 0), (2 2 4) and (3 1 4) degrees, respectively^24^. The ZnO phase was ascribed to the peaks at 54.77° and 65.45°. (JCPDS no: 36-1451). NiS phase was assigned to the peaks at 30.2°, 34.8°, 37.9°, 53.5°, and 73.32°. (JCPDS no: 02-1280)^23,25,26^.

**Figure 2:**
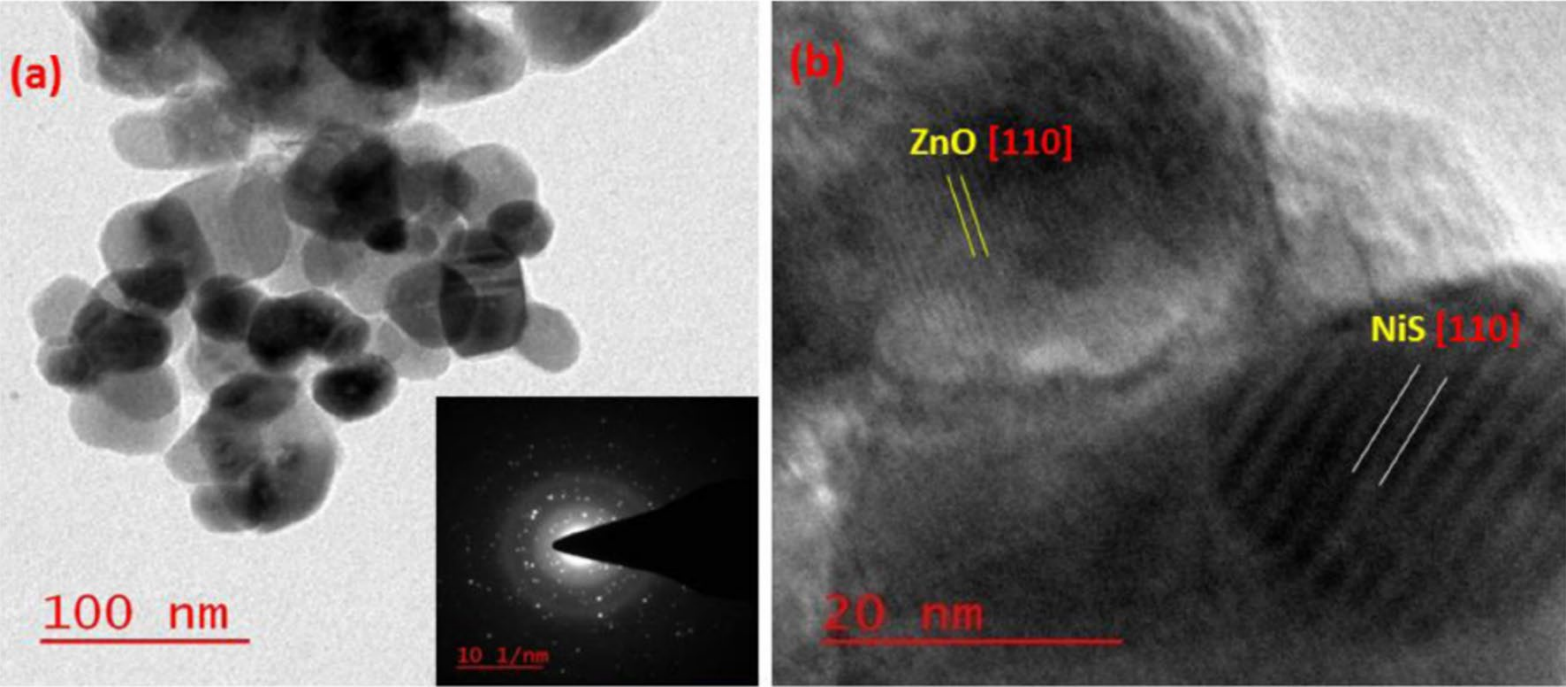
(**a**) TEM micrograph of NiS/ZnO (inset showing SAED), (**b**) HR-TEM of NiS/ZnO.

The morphological behaviour of the produced nanoparticles is depicted in TEM micrographs. The nearly spherical TEM picture of the NiS/ZnO nanocomposite is shown in Fig. 2a, which is very crystalline. HR-TEM of NiS/ZnO indicates a tight interfacial interaction between ZnO and NiS nanoparticles, as shown in Fig. 2b. The matching d-spacing value of the image confirms the fringes of a lattice. For ZnO and NiS, the computed d-spacing was discovered to be 0.15 nm and 0.48 nm, corresponding to the [110] plane for both.

### Evaluation of photocatalytic measurements

It is difficult to predict wavelength-dependent photochemical reactivity. Here, we resurrect a tried-and-true voltage measurement tool and modify it to map LED wavelengths. This study presents a practical methodology for enhancing charge transport by integrating NiS/ZnO into perovskite and, as a result, a straightforward modification procedure to create high-performance mPSCs. Herein, an NI source meter was used to measure the voltage generated by the photochemical activity.

### Selectivity analysis

For the selectivity analysis, voltage measurement was carried out using different light sources (UV light-333 nm, 365 nm, 400 nm, 420 nm, 430 nm, 450 nm, 510 nm and 557 nm). The initial delay of the light source was 30 seconds, and it remained on for two minutes.

**Figure 3:**
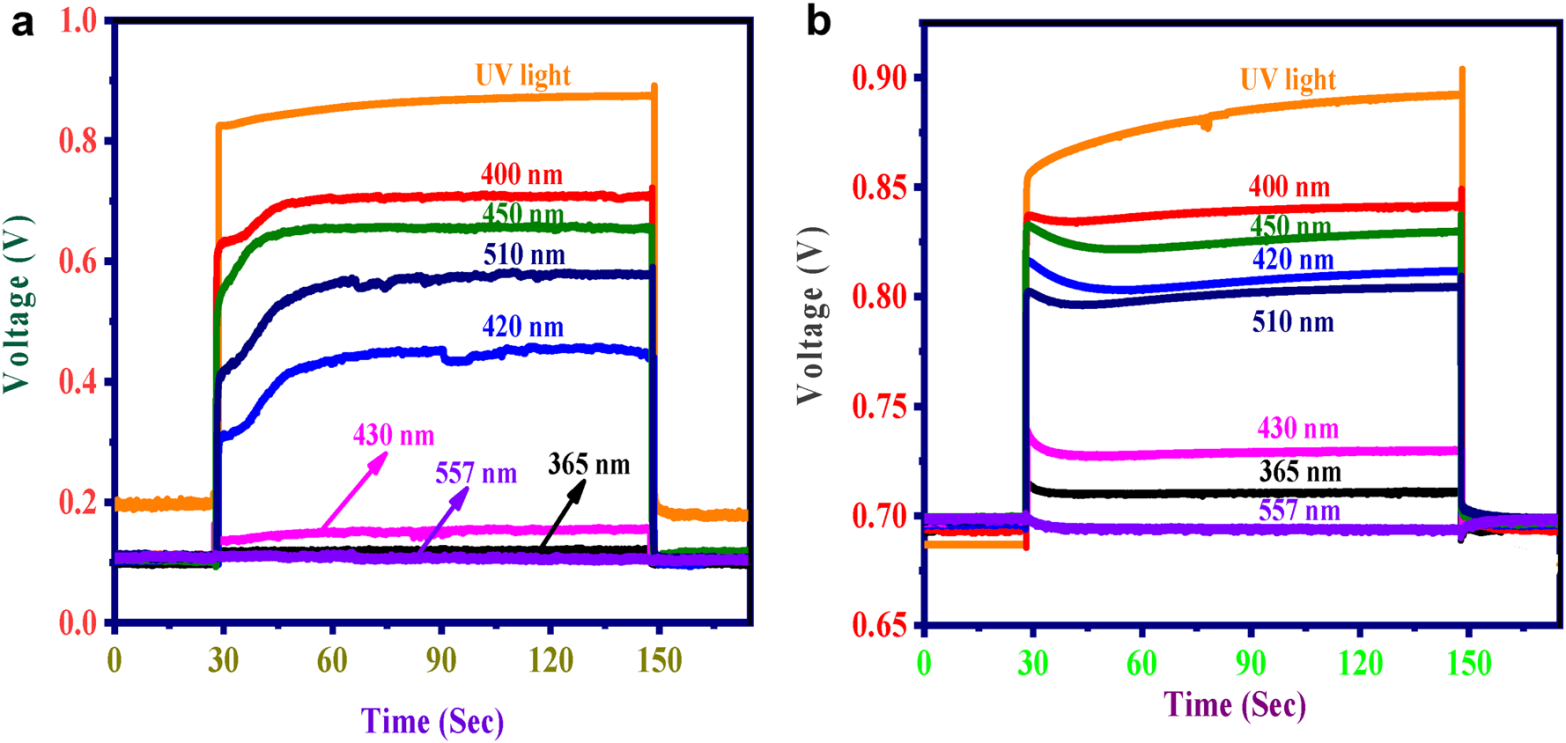
Selectivity analysis (**a**) perovskite alone, (**b**) NiS/ZnO with perovskite.

A high degree of selectivity can be achieved by controlling the photo-kinetics by tuning the wavelength of employed LEDs between 365 and 557 nm (Fig. 3). It was observed that each wavelength produces unique voltage during analysis which makes the system highly selective and specific. It was also evident from the analysis that NiS/ZnO (Fig. 3b) produces more voltage compared to perovskite alone (Fig. 3a). This study demonstrates how adding a NiS/ZnO composite to perovskite solar cells significantly changes their absorbance and reactivity, enabling very wavelength-selective photoreactions. This discovery supports the significance of the work done in precision photochemistry, where the careful characterization of reactive substances and the well-thought-out design of experiments support the development of sophisticated applications.

### Stability analysis

**Figure 4:**
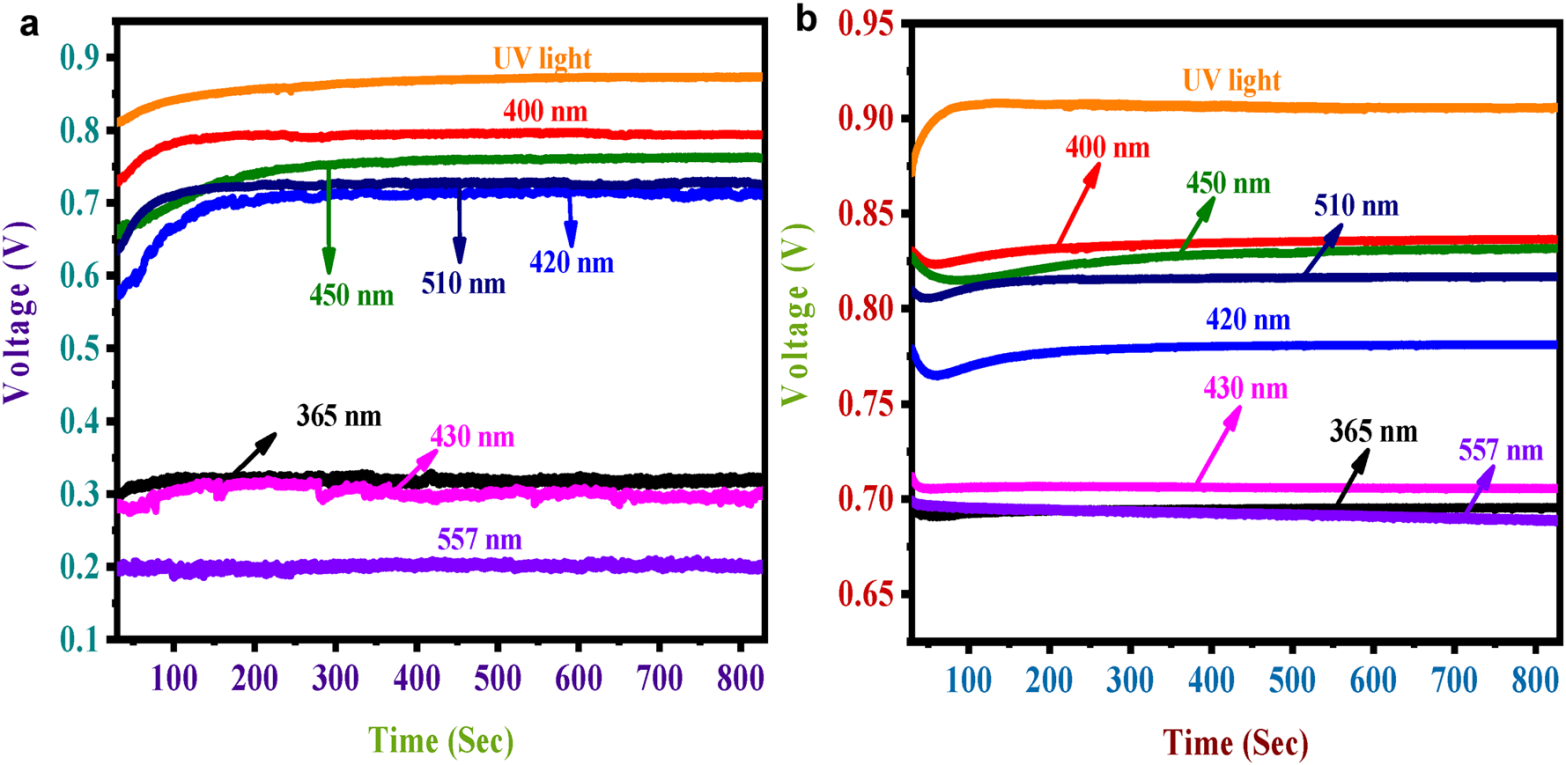
Stability analysis (**a**) perovskite alone, (**b**) NiS/ZnO with perovskite.

The light source was kept off for 30 seconds for the stability analysis. Afterward, for the next 800 seconds, it was on. Analysis was carried out for the perovskite solar electrode and NiS/ZnO modified electrode. Figure 4 illustrates how NiS/ZnO's photocatalytic activity did not significantly decline over time, indicating the photocatalysts showed high photochemical stability. Studies like these, which concentrate on photostability, aid in identifying appropriate photocatalysts for various applications, just as the stability of photocatalytic processes has been a problem and troubled the industry for a while.

### Sensitivity analysis

**Figure 5:**
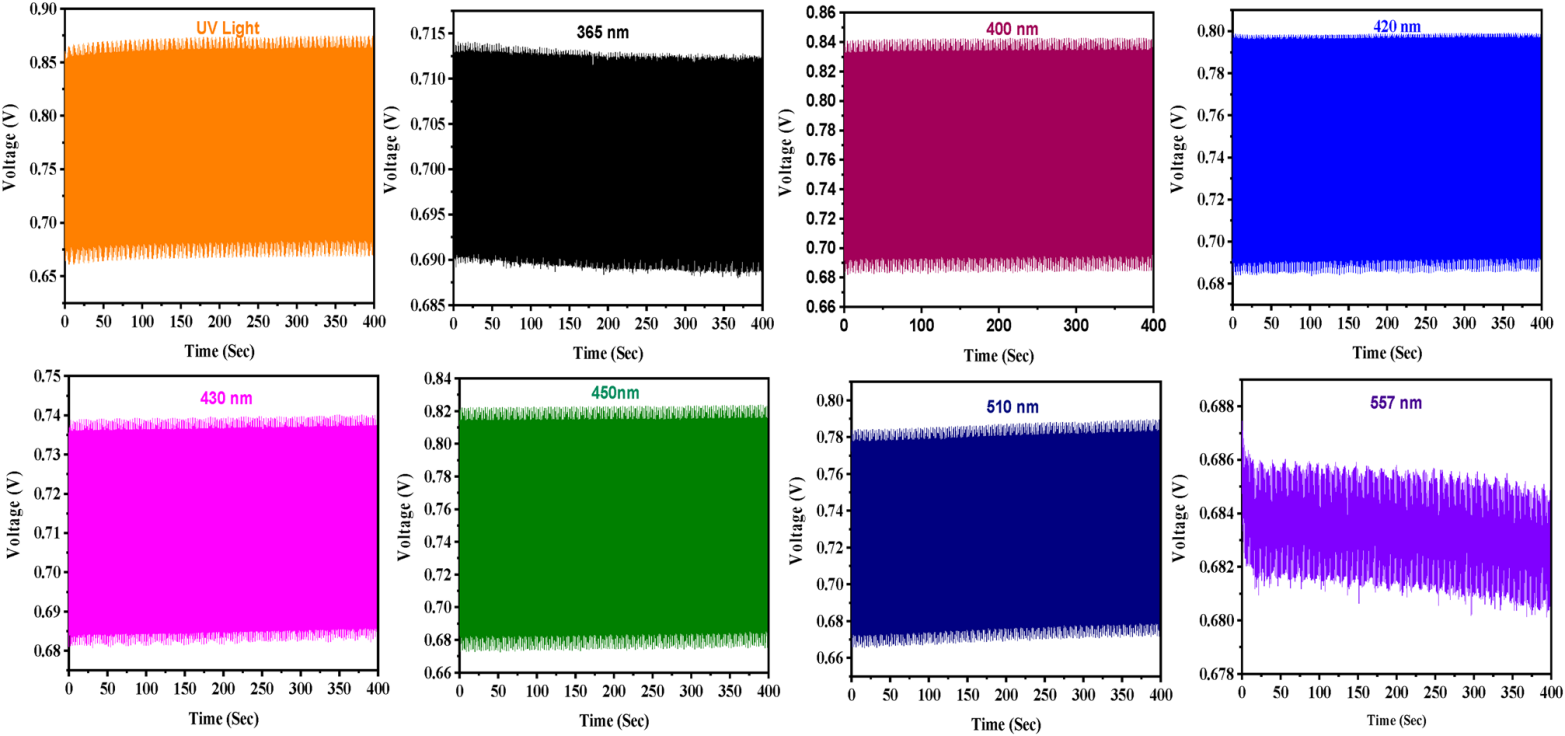
Sensitivity analysis of NiS/ZnO modified perovskite electrode with different LEDs.

To examine the reusability and sensitivity of the nanocomposite-modified solar electrodes, the photocatalytic experiment was carried out by keeping the light source off for two seconds and then on for two seconds and was repeated for 400 s. The result of the recyclability test is displayed in Fig. 5, which indicates that nearly all LEDs apart from 557 nm exhibited excellent durability. In the case of 557 nm LEDs, a slight decrement is observed after 250 seconds.

### Voltage enhancement by NiS/ZnO composite

**Figure 6:**
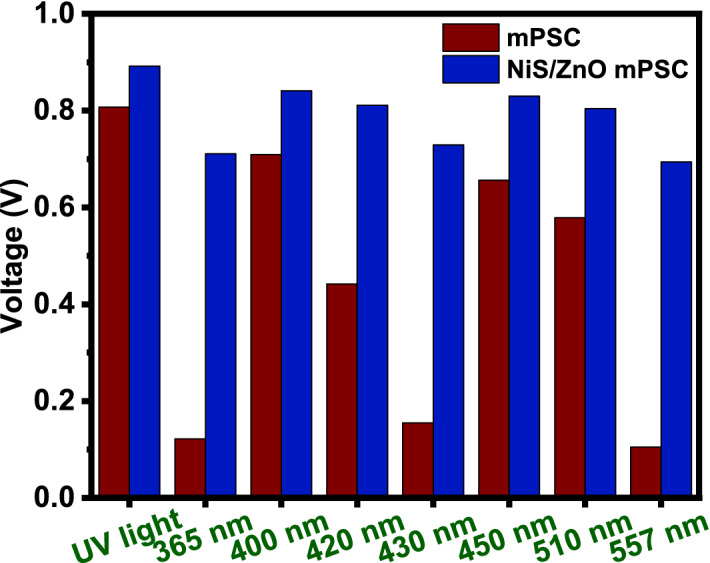
Voltage enhancement by NiS/ZnO modified perovskite electrode with different LEDs.

Figure 6 represents the voltage enhancement achieved through modifying perovskite solar cells. In the case of 365, 450 and 557 nm LEDs, voltage enhanced drastically after the perovskite was modified with NiS/ZnO composite. Other LEDs also exhibited an increment in voltage when the electrode was modified.

### Humidity and temperature effect

Smart monitoring, management, and control of interior settings is one potential sensor application area. Using common sources and simultaneous measurement, we performed an optimal matrix of tests under various temperature and relative humidity (RH) circumstances in this study.

**Figure 7:**
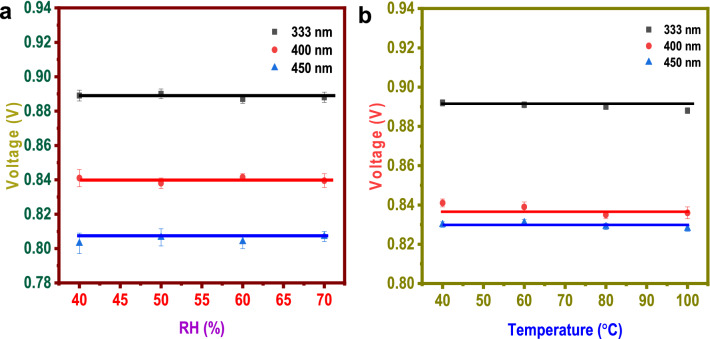
(**a**) Humidity effect, (**b**) temperature effect.

This work performed a series of tests where temperature and RH change in a perfectly controlled environment. For the analysis, we chose three different LEDs (333 nm, 400 nm and 450 nm, which produced higher voltage). This study evaluated the effect of four relative humidity environments, i.e., 40%, 50%, 60% and 70% RH, at a constant temperature. The analysis observed no change in photocatalytic activity in the four relative humidity environments (Fig. 7a).

To evaluate the effect of temperature on the sensing environment, measurement was carried out at four different temperatures, 40 °C, 60 °C, 80 °C and 100 °C. No statistically significant correlation between temperature and low-cost sensor output was found for any sensor evaluated in the range of 40–100 °C, indicating that this variable is likely not a significant factor in the deterioration of sensor performance in indoor environments. (Fig. 7b).

## Conclusion

An effective design for photochemically selective reaction systems was developed in the current research, which is also essential for wavelength prediction in applications like photolithography. NiS/ZnO nanocomposite was prepared using a straightforward hydrothermal process. To enhance the functionality of mPSCs, we clarified the potential role of NiS/ZnO as an addition to the MAPbI_3_ perovskite structure. The simple method produced superior photoactive films with fewer fault states. The photocatalytic activity of the prepared samples was monitored under exposure to different light sources. These modified mPSCs were highly specific in predicting the wavelength of different LEDs. This research offers a comprehensive view of photochemical activity's potential and its recently developed uses in creating wavelength-selective photosensors and for implementing in photolithography.

## Methods

### Materials

Analytical reagent (AR) grade zinc acetate dihydrate (Zn(CH_5_CO_3_)_2_·2H_2_O) (Sigma-Aldrich), Nickel chloride hexahydrate (NiCl_2_·6H_2_O) (Sigma-Aldrich), sodium thiosulfate pentahydrate (Na_2_S_2_O_3_·5H_2_O) (Spectrum), potassium hydroxide (KOH) (Merck), sodium hydroxide (Merck), N,N-Dimethylformamide (Alfa Aesar), MAPbI_3_ (Solaronix SA) were utilized without further purification.

### Preparation of the ZnO

In this process, 2 M of the NaOH is dissolved in 20 ml of distilled water and added dropwise to 0.5 M of zinc acetate dihydrate (Zn(CH_5_CO_3_)_2_·2H_2_O) dissolved in 50 ml of distilled water and stirred for 15 minutes at room temperature. The white precipitate was transferred into 100 ml of autoclave and maintained the hydrothermal temperature at 160 °C for 5 hours. The same further procedure was followed for preparing the NiS/ZnO nanocomposite.

### Preparation of the NiS/ZnO composite

**Figure 8:**
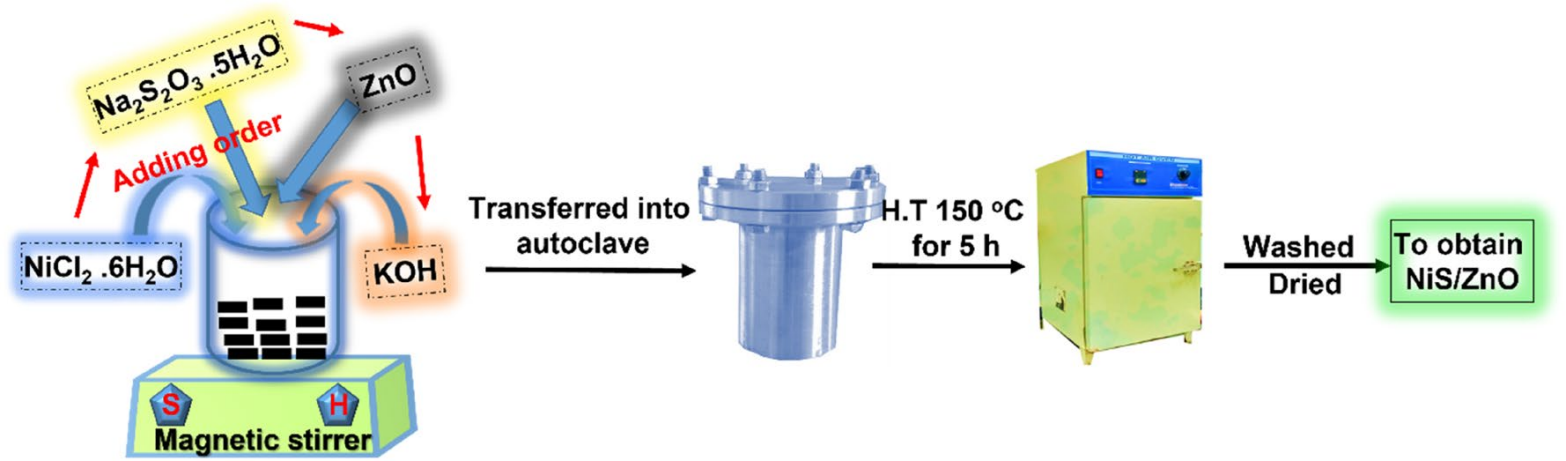
Fabrication of NiS/ZnO composite.

In this procedure, 2.3769 grams of the nickel chloride hexahydrate (NiCl_2_.6H_2_O) was dissolved in 40 ml of distilled water and then combined with 100 mg of ZnO and 0.5 M of 20 ml sodium thiosulfate pentahydrate (Na_2_S_2_O_3_·5H_2_O) added to above solution mixture stirred continuously for 10 minutes at room temperature. Further, added 1 M of 10 ml potassium hydroxide (KOH) to the above mixture dropwise and stirred for 15 minutes at room temperature. The hydrothermal temperature is then maintained at 150 °C for 5 hours in a 100-cc autoclave while kept in a hot air oven. To get NiS/ZnO nanocomposite, the reaction autoclave's end was cleaned with three times water and one-time ethanol, then dried at 100 °C overnight (Fig. 8).

### Fabrication of solar cell

The printed electrodes were by Solaronix SA (Aubonee, Switzerland) and were made of glass, FTO, compact TiO_2_, mesoporous TiO_2_, mesoporous ZrO_2_, and monolithic carbon. The perovskite solution, MAPbI_3_ (a combination of lead iodide, methylammonium iodide, and 5-aminovaleric acid hydroiodide), was mixed with 5% of 0.1 M NiS-ZnO in DMF (N,N-Dimethylformamide) solvent. The mesoporous structure was coated with 0.5 µl of the prepared precursor solution. The prepared films were annealed for 30 minutes at 100 °C in an ambient environment.

### Characterisation

Under Cu K radiation, the crystal structures of all the samples were examined using a Bruker D8 Advance X-ray diffractometer (XRD). The morphologies and element analyses were seen using JEM-2100 high-resolution transmission electron microscopy.

### Measurement of photocatalytic activity

A photo-assisted voltage measurement utilizing NiS/ZnO mPSC was used to assess the photocatalytic effectiveness of the produced catalysts. The photocatalytic experiment was carried out using UV-light and LEDs having different wavelengths (365, 400,420, 430, 450, 510 and 557 nm) as an irradiation source. Light from these irradiation sources was made to fall on the solar electrode, which is connected to an NI source meter. This instrument measures voltage against time. Selectivity, stability and sensitivity were also measured. For practical application, a sensor was developed using Arduino (Microcontroller), LCD display, Bread board and Jumper wires (Supplementary files).

## Supplementary Information

Supplementary Legends.

Supplementary Video 1.

## Data Availability

The data that support the findings of this study are available on the request from the corresponding author.
