# Supplementary material for: A practical perspective for chromatic orthogonality for implementing in photolithography
Source: Sci Rep. 2023 Jan 13;13:694. doi: 10.1038/s41598-023-27869-w (PMC9839670; doi:10.1038/s41598-023-27869-w)
Supplement: Supplementary file 1 — Supplementary Legends. [file 41598_2023_27869_MOESM1_ESM.docx]

For the practical application of the developed method, we have fabricated a setup as shown in the video. Inside the setup, an electrode coated with NiS/ZnO is placed. LEDs are projected towards this electrode, and the voltage of the LEDs can be measured in this prototype. Asper the value of voltage, the prototype can predict the wavelength of LEDs. For the video, e have kept 420nm LED, and the prototype was successful in predicting the wavelength.
